# Hereditary hemorrhagic telangiectasia and COVID-19

**DOI:** 10.1590/0037-8682-0785-2020

**Published:** 2020-11-25

**Authors:** Rachel Zerbini Mariano, Monica Corso Pereira, Fabiano Reis

**Affiliations:** 1 Universidade Estadual de Campinas, Faculdade de Ciências Médicas, Departamento de Radiologia, Campinas, SP, Brasil.; 2 Universidade Estadual de Campinas, Faculdade de Ciências Médicas, Departamento de Clínica Médica, Campinas, SP, Brasil.

**FIGURE 1:**
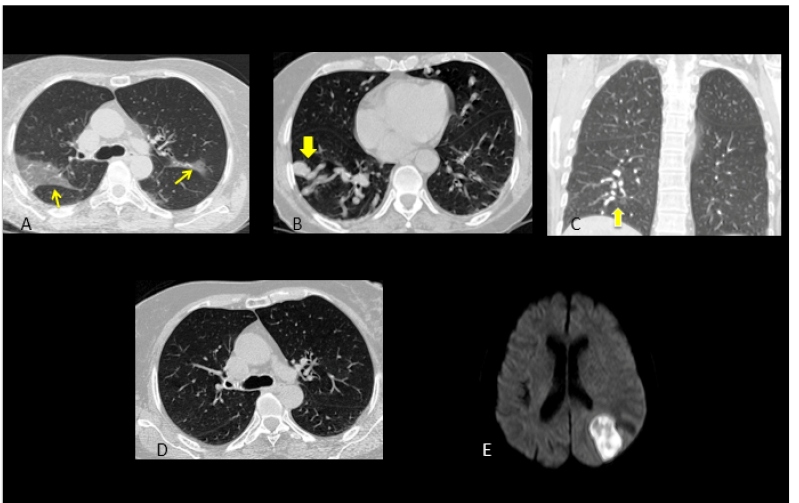
(A) Chest computed tomography, pulmonary window, axial view, showing bilateral multifocal areas of mainly peripheral consolidations (arrows). Chest computed tomography, pulmonary window, axial (B) and coronal (C) views, demonstrating multiple dilated and tortuous vessels consistent with pulmonary arteriovenous malformation (arrow). (D) Chest computed tomography, pulmonary window, axial view, performed 1 month earlier and showing no ground-glass opacities. (E) - Axial magnetic resonance imaging (diffusion-weighted imaging) showing restricted diffusion in the central content of a left parietal lesion, a finding consistent with pyogenic abscess.

A 65-year-old woman with hereditary hemorrhagic telangiectasia (HHT) for which she was on home oxygen therapy without medication intake, was admitted to our emergency department for fever and worsening dyspnea. Her oxygen saturation on admission was 90%, akin to that at baseline. A Chest computed tomography (CT) scan revealed peripheral consolidations (Figure 1A) and enlarged feeding arteries, nodules, and draining veins (Figures 1B and 1C); nonetheless, a CT scan performed 1 month earlier showed no consolidations (Figure 1D). Real-time polymerase chain reaction confirmed SARS-CoV-2 infection. Despite early hospitalization and oxygen therapy by catheter and orotracheal intubation, the patient showed a progressive worsening of the respiratory function, developed a brain abscess (Figure 1E), and died 26 days following admission.

HHT is an autosomal dominant disease, and the diagnostic criteria are the presence of epistaxis, multiple telangiectasias on mucocutaneous surfaces, arteriovenous malformations (AVMs) in larger organs, and a family history of the disease^1^ ^,^ ^2^. Oxygenation is commonly affected in patients with HHT^1^ ^,^ ^2^, and the frequent manipulation of the nostrils, nasal cavity, or nasopharynx may predispose them to contracting Coronavirus Disease 2019 (COVID-19)^3^. Moreover, patients with HHT have comorbidities that may negatively influence COVID-19 outcome, such as chronic anemia, heart failure, pulmonary AVMs, pulmonary hypertension, and chronic hypoxemia^3^.

Patients with COVID‐19 are predisposed to developing thrombosis, which may lead to thrombotic complications in a context of HHT^3^. Although HHT is rare, it deserves special attention in patients with COVID‐19.
